# Development of a small cell lung cancer organoid model to study cellular interactions and survival after chemotherapy

**DOI:** 10.3389/fphar.2023.1211026

**Published:** 2023-08-07

**Authors:** Chandani Sen, Caroline R. Koloff, Souvik Kundu, Dan C. Wilkinson, Juliette M. Yang, David W. Shia, Luisa K. Meneses, Tammy M. Rickabaugh, Brigitte N. Gomperts

**Affiliations:** ^1^ Department of Pediatrics, David Geffen School of Medicine, UCLA Children’s Discovery and Innovation Institute, Mattel Children’s Hospital, University of California, Los Angeles, CA, United States; ^2^ Intel Labs, San Diego, CA, United States; ^3^ Pulmonary Medicine, David Geffen School of Medicine, University of California, Los Angeles, CA, United States; ^4^ Jonsson Comprehensive Cancer Center, University of California, Los Angeles, CA, United States; ^5^ Eli and Edythe Broad Stem Cell Research Center, University of California, Los Angeles, CA, United States

**Keywords:** cancer organoids, lung fibroblast, 3D model, phenotypic tool, chemotherapy, survival, conditioned media, image processing

## Abstract

**Introduction:** Small-cell-lung-cancer (SCLC) has the worst prognosis of all lung cancers because of a high incidence of relapse after therapy. While lung cancer is the second most common malignancy in the US, only about 10% of cases of lung cancer are SCLC, therefore, it is categorized as a rare and recalcitrant disease. Therapeutic discovery for SCLC has been challenging and the existing pre-clinical models often fail to recapitulate actual tumor pathophysiology. To address this, we developed a bioengineered 3-dimensional (3D) SCLC co-culture organoid model as a phenotypic tool to study SCLC tumor kinetics and SCLC-fibroblast interactions after chemotherapy.

**Method:** We used functionalized alginate microbeads as a scaffold to mimic lung alveolar architecture and co-cultured SCLC cell lines with primary adult lung fibroblasts (ALF). We found that SCLCs in the model proliferated extensively, invaded the microbead scaffold and formed tumors within just 7 days. We compared the bioengineered tumors with patient tumors and found them to recapitulate the pathology and immunophenotyping of the patient tumors. When treated with standard chemotherapy drugs, etoposide and cisplatin, we observed that some of the cells survived the chemotherapy and reformed the tumor in the organoid model.

**Result and Discussion:** Co-culture of the SCLC cells with ALFs revealed that the fibroblasts play a key role in inducing faster and more robust SCLC cell regrowth in the model. This is likely due to a paracrine effect, as conditioned media from the same fibroblasts could also support this accelerated regrowth. This model can be used to study cell-cell interactions and the response to chemotherapy in SCLC and is also scalable and amenable to high throughput phenotypic or targeted drug screening to find new therapeutics for SCLC.

## 1 Introduction

Lung cancer is the largest cause of cancer death in both men and women worldwide (cancer.net, 2022), with an overall 5-year survival rate of only about 23%, according to the Surveillance, Epidemiology, and End Results (SEER) data SEER, (2022). However, this overall 5-year survival rate decreases to 8% if distant tumor spread is present at diagnosis. Of all the lung cancer subtypes, small cell lung cancer (SCLC), a neuroendocrine sub-type representing about 10% of all lung cancers, has by far the worst prognosis and is often highly metastatic (Stovold et al., 2013; Byers and Rudin, 2015; Raso et al., 2021). The classic neuroendocrine markers (synaptophysin, chromogranin A, and NCAM) are usually used for diagnosis (Miyauchi et al., 2015) of the tumor biopsy sample and the cisplatin-etoposide combination is used as standard first line chemotherapy (Byers and Rudin, 2015). The main cause of death in these patients is resistance to chemotherapy as most patients respond well to initial therapy but will experience tumor recurrence within 1 year after completing chemotherapy (Shia et al., 2022). The underlying pathogenesis of this tumor resistance is still unknown and there have been no advances in therapies for more than three decades (Sabari et al., 2017).

Drug screening for cancer is usually performed initially with cancer cell lines grown in aggregates in plastic dishes. These cells are easy and cheap to grow and convenient for high throughput screening, but they fail to represent the complexity of the tumor microenvironment (Sen et al., 2022). These kinds of cancer models show about a 10% success rate in developing anti-cancer drugs for clinical trials. But because it is challenging to model relapse in SCLC, most of the drugs identified failed at preventing recurrence in pre-clinical and clinical trials (Martinez-Pacheco and O’Driscoll, 2021). The current gold standards are patient-derived xenografts (PDX), where small pieces of tumor tissue derived from patients’ are transferred to immune-deficient mice and cell line-derived xenografts (CDX) (Simpson et al., 2020), where cancer cell lines are transferred to immune-deficient mice to predict drug efficacy. But these models also have several limitations, including chances of tumor tissue engraftment failure, a long tumor development timeline, dissimilarity of the tumor microenvironment between human and murine models, and low throughput for drug screening (Jung et al., 2018). An alternative model for animal PDX/CDX modeling will also address the 3Rs’ (replacement, refinement and reduction) that are essential for animal welfare (Kirk, 2018). Recently, 3D human organoid/spheroid models are gaining popularity in cancer research (Truong et al., 2016, Klameth et al., 2017, Kuriakose et al., 2019, Ramamoorthy et al., 2019, (Ma et al., 2022), (Wang et al., 2020) but currently there is no existing human co-culture SCLC organoid model to study the tumor microenvironment and phenotypic changes after chemotherapy that can be useful in drug screening for SCLC relapse.

To address these issues, we have developed a scaffold-based 3D organoid model mimicking lung micro-architecture using primary human healthy adult lung fibroblasts (ALF) and SCLC cell lines. This cell co-culture organoid model shows phenotypic changes consistent with disease progression *in vitro* and allows the assessment of the role of the fibroblasts in the development of disease recurrence after chemotherapy. In addition, our model is scalable for 96-well and 384-well plates and therefore is valuable for high throughput screening (HTS) for therapeutic strategies to prevent SCLC relapse.

## 2 Results

### 2.1 The co-culture SCLC organoid model recapitulates tumor growth, local invasion and the mechanobiology of SCLC

**FIGURE 1:**
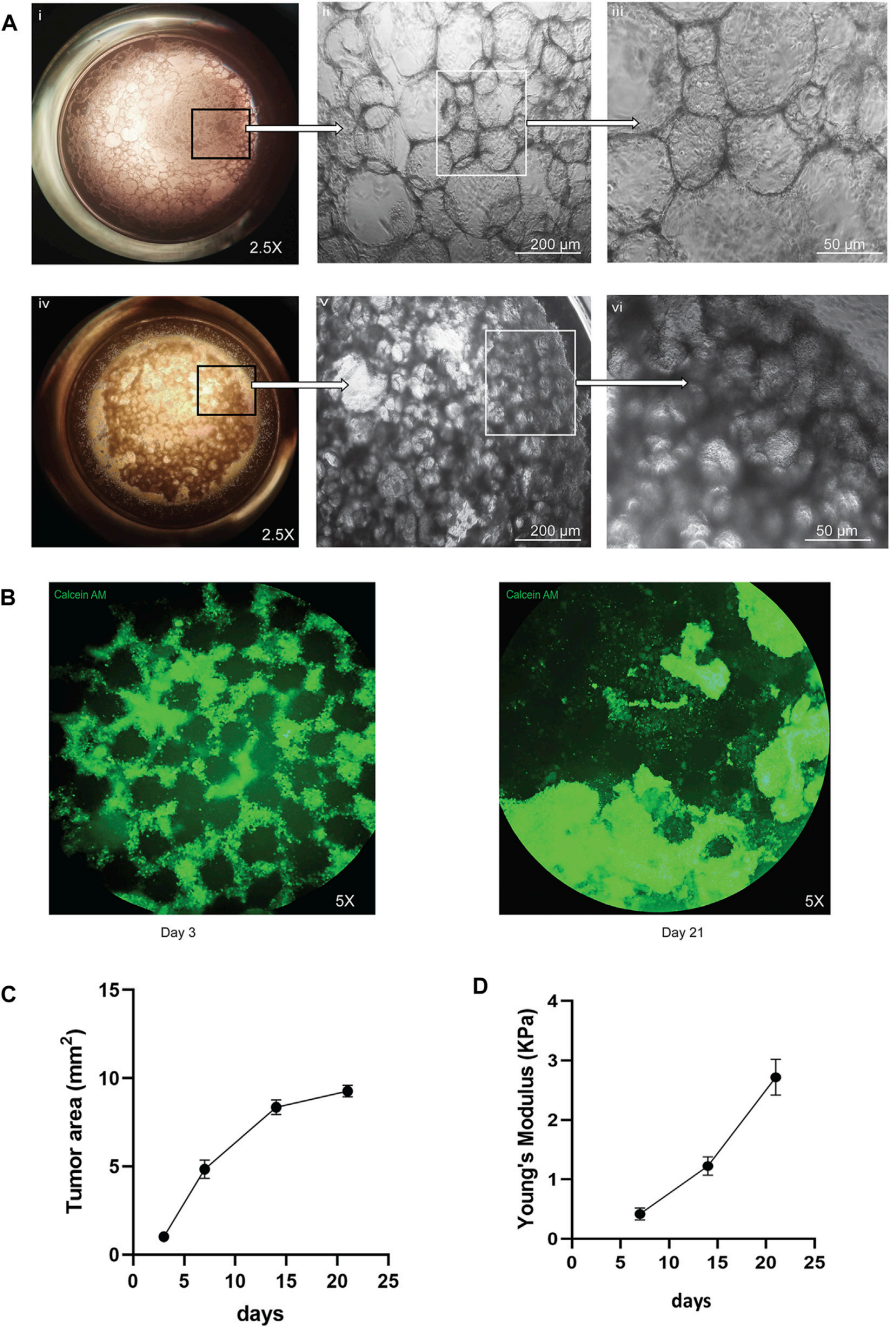
Visual representation of the co-culture SCLC (H526) organoid model: **(A)** Difference in healthy (ALF only) and cancer (SCLC-ALF) organoid phenotype after 72 h: **(Ai–iii)** ALF only organoid model at 2.5X, 10X, and 20X magnification, **(Aiv–vi)** SCLC-ALF co-culture organoid model at 2.5X, 10X, and 20X magnification. **(B)** Change in SCLC-ALF organoid phenotype between culture day 3 and day 21. **(C)** Tumor growth kinetics as observed by Calcein-AM (live cell dye)-stained organoid area measurement. **(D)** Plot of Young’s modulus of whole organoid model shows the increase in organoid stiffness over time in culture.

To recapitulate the 3D lung alveolar micro-architecture that small cell lung cancer (SCLC) grows in, we developed a microbead based tumor organoid model that was scaled to a 96-well plate. First, we generated alginate microbeads using an electrostatic droplet generator and then coated the beads with ALFs from patients with no history of prior lung disease or lung cancer. Figures 1Ai–iii demonstrates the growth of ALFs only on the microbead scaffold. Then, to recapitulate the SCLC cell growth and disease initiation in a healthy lung, we added different ratios of ALF’s: SCLC cells. We found that the combinations of ALF’s: SCLC of 1:1 or 1:4 became rapidly overcrowded by the growth of the SCLC cells (Supplementary Figure S1A). We therefore first added ALF’s to the microbeads as the main cell population (80% of total cells) using the ALF media for 24 h, and then introduced SCLC cells (20% of total cells) in the system using the co-culture media (mixture of 80:20 ALF: SCLC media). Figures 1Aiv–vi shows this ALF: SCLC 4:1 co-culture model and demonstrates how the SCLC cells proliferated and invaded the ALF coated microbeads with a visible difference in phenotype compared to the 3D cultures that consisted only of ALFs. In order to better visualize these two cell populations, we used a vimentin promoter RFP reporter for the ALFs and an EpCAM promoter GFP reporter to visualize the SCLC cells because the SCLC cells are the only epithelial cells present in the model. Supplementary Video S1 shows a 3D rendering of a whole SCLC-ALF co-culture organoid model with the location of these cell types. To monitor the SCLC phenotype over time, we stained the organoid models with the live cell permeant dye Calcein AM and imaged them at days 3–21 of culture (Figure 1B). Initially, we noticed SCLC cells and fibroblasts surrounded the scaffolds (Figure 1B, day 3) but between days 7–21 of culture, large SCLC cell clusters formed which took over the cultures and completely displaced the scaffolds showing an invasive tumor phenotype (Figure 1B, day 21). Supplementary Video S2 shows brightfield imaging of the movement of SCLC cells surrounding the microbeads in the model microenvironment. We next calculated the tumor area in the 3D SCLC co-culture organoid model using ImageJ, a Java based imaging program from the National Institutes of Health. We found a 9-fold increase in tumor area over days 3–21 in the SCLC organoid after which growth plateaued in the cultures (Figure 1C), which likely occurs due to physical microenvironmental constraints. We tested the efficacy of our model to recapitulate the SCLC growth and phenotype with three SCLC cell lines- H526, H1963, and H82, each in combination with ALF’s. All cell lines showed a similar pattern of tumor growth. The brightfield images of the culture phenotypes are shown in Supplementary Figure S2.

Tissue mechanics, including tissue stiffness, provide physical cues that are a vital microenvironmental factor that can affect cell behavior. We measured the physical stiffness of the cells in our SCLC co-culture organoid model over time in culture. We used JPK Nanowizard 4a Atomic Force Microscopy to show that the change in stiffness in terms of Young’s Modulus was increased about 5-fold from day 7 to day 21 of culture, as shown in Figure 1D. This progressive increase in cell stiffness may result from increasing cell-cell and cell-extracellular matrix (ECM) interactions as the tumor grows over time. Taken together, this SCLC-ALF co-cultured organoid model recapitulates rapid 3D tumor growth, the invasive behavior of SCLC, and mechanobiological changes seen in SCLC. We therefore next sought to validate the SCLC organoid model by assessing the ability of the organoid to produce the neuroendocrine markers seen in SCLC tumors.

### 2.2 The SCLC co-cultured organoid model produces classic neuroendocrine markers of SCLC tumors

**FIGURE 2:**
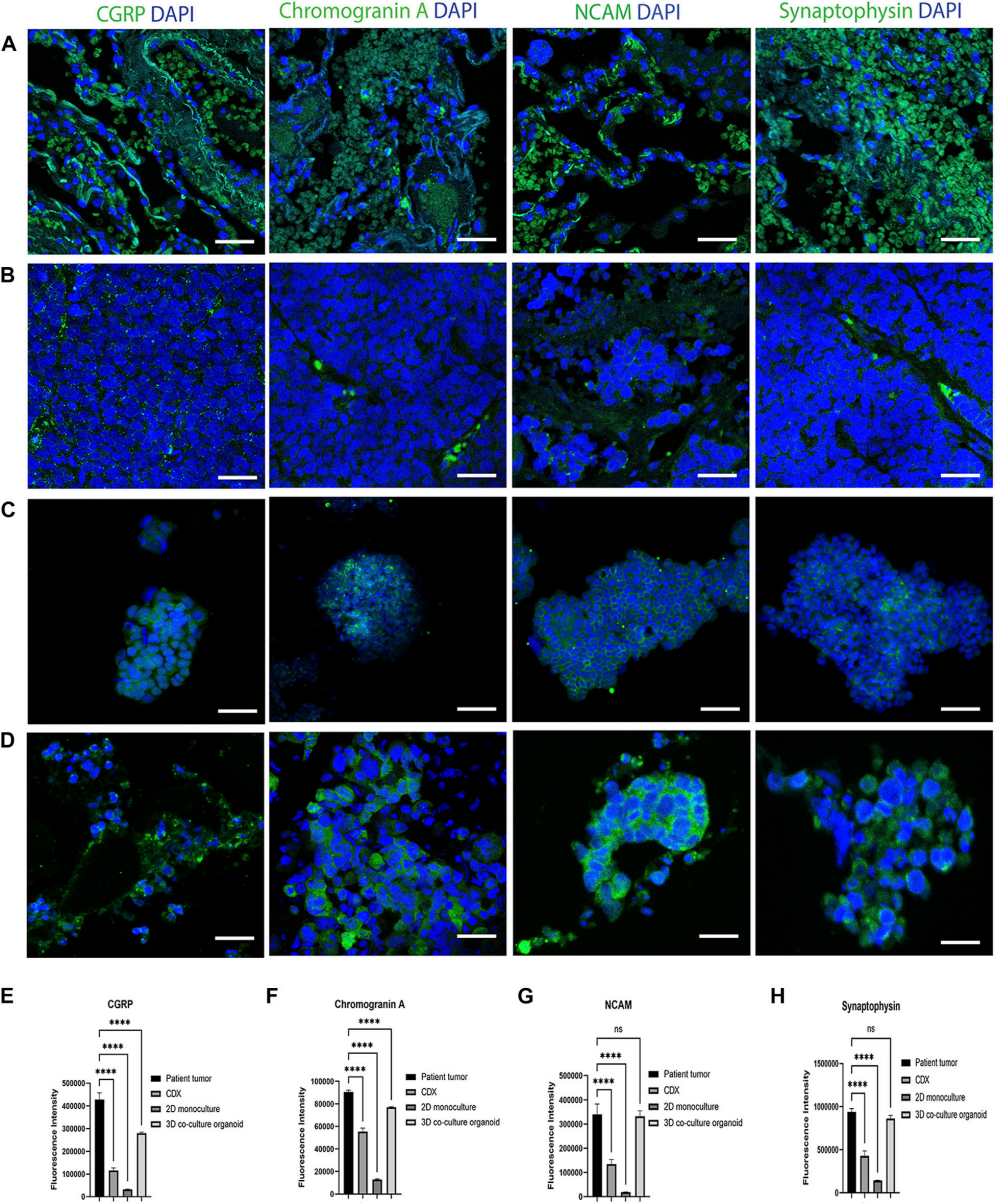
SCLC classic neuroendocrine biomarkers are expressed in the SCLC co-culture organoid model: CGRP, Chromogranin A, NCAM and Synaptophysin expression in **(A)** patient tumor; **(B)** cell line-derived xenograft (CDX), **(C)** SCLC cell line in 2D monoculture and **(D)** SCLC co-culture 3D organoid tumor. CDX, SCLC monoculture and SCLC co-culture organoid were developed from the same SCLC cell line (H526). The scale bar is 20 µm. **(E–H)** Fluorescence intensity quantification plots of all models: **(E)** CGRP, **(F)** Chromogranin A **(G)** NCAM, and **(H)** Synaptophysin. Bar graph represents SEM; n = 3. ∗∗∗∗*p* < 0.0001 by one-way ANOVA.

SCLC is the most common form of neuroendocrine lung cancer and produces the classic neuroendocrine markers, Chromogranin A, synaptophysin, NCAM (CD56) and Calcitonin gene-related peptide (CGRP), which are commonly used as SCLC tumor markers (Stovold et al., 2013; Martinez-Pacheco and O’Driscoll, 2021). To further validate the co-cultured organoid model’s pathophysiological characteristics, we compared the expression of CGRP, Chromogranin A, NCAM and Synaptophysin in the patient SCLC tumor sections (Figure 2A), cell-culture derived xenografts (CDX) tumors (Figure 2B), 2D SCLC cell line cultures (Figure 2C) and in the developed organoid model (Figure 2D) at 14 days of culture. The CDX tumors and 2D cell cultures were developed from the same SCLC cell line (H526) as was used to generate the SCLC-ALF co-cultured organoid model.

The corresponding fluorescence intensity plots for CGRP, Chromogranin A, NCAM and synaptophysin are in Figures 2E–H that confirms that the SCLC patient tumor displayed the highest expression of all 4 biomarkers tested. The 3D co-cultured organoid model demonstrated the second highest expression followed by the CDX tumor and the 2D SCLC cell line cultures. This result elucidates that the cells within the SCLC organoid model express marker proteins characteristic of SCLC tumors and therefore we next sought to examine the response of the SCLC organoid model to chemotherapy.

### 2.3 SCLC co-culture organoid model demonstrates greater tumor cell survival and recurrence after chemotherapy than the SCLC monoculture organoid model

As the SCLC-ALF co-culture organoid models demonstrate several key features of the disease, we tested their ability to model the relapse of SCLC after chemotherapy, which is the most challenging aspect of this tumor. For this we used the standard combination chemotherapy of Cisplatin and Etoposide at the half maximal inhibitory concentration (IC50). IC50 values were calculated for three SCLC cell lines in the model in the co-culture setting and the values are in Supplementary Table S1. We then chose to continue the next experiments with one representative SCLC cell line (H526).

**FIGURE 3:**
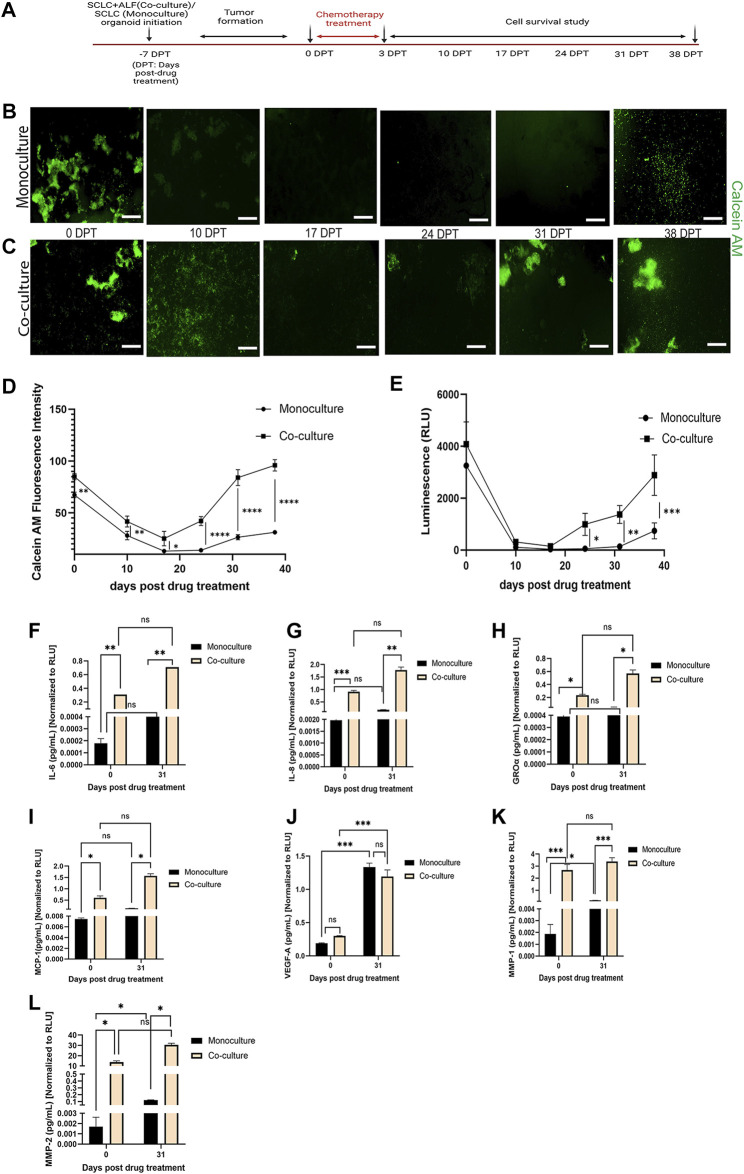
SCLC co-culture organoid model recapitulates tumor cell survival and recurrence post-chemotherapy more closely than SCLC monoculture organoid model: **(A)** experimental timeline. Fluorescent images of cell survival after chemotherapy as observed by Calcein AM (live cell dye)-stained **(B)** monoculture organoid model of SCLC cells and **(C)** co-culture organoid model of SCLC cells and ALFs (scale bar: 500 µm). Cell survival and regrowth in the model plotted by **(D)** Calcein AM fluorescence intensity measurement and **(E)** Cell-titre glo cell viability assay. Data point on the plots represents SEM; n = 3. ∗*p* < 0.05, ∗∗*p* < 0.01, ∗∗∗*p* < 0.001 by two-way ANOVA multiple comparison test. Quantification of acute inflammatory factors secreted by monoculture and co-culture organoid: **(F–G)** cytokines, **(H–J)** chemokines and **(K–L)** MMPs before drug treatment (0 DPT) and after regrowth of cells (31 DPT). Secreted factor values were normalized against RLU values (0 and 31 DPT) of mono- and co-culture organoid before plotting. Bar graph represents SEM; n = 3. ∗*p* < 0.05, ∗∗*p* < 0.01, ∗∗∗*p* < 0.001 by two-way ANOVA multiple comparison test. The H526 cell line was used as the SCLC population in this experiment.

In order to better understand the role of ALFs in the SCLC cell’s response to chemotherapy, in addition to the co-culture model, we also generated monoculture organoid models containing only SCLC (H526) cells, in monoculture media. Both mono- and co-cultured SCLC organoid models were treated with the IC50 dose of Cisplatin and Etoposide and monitored for cell survival. A brief workflow of this chemotherapy exposure experiment is shown in Figure 3A.

We found that both the SCLC mono- and co-cultured organoid model tumors formed within 7 days of culture. We then added Cisplatin and Etoposide (labeled 0 days post treatment (0 DPT)) at their combined IC50 dosage into the respective mono- and co-culture organoid media. After 72 h, we removed the Cisplatin and Etoposide and monitored the organoid models weekly for any change in tumor phenotype. For that, we measured live cell dye Calcein AM uptake (live cell fluorescence intensity, reflecting a change in live cell number) (Figure 3B) by automated image analysis using Otsu’s method (Otsu, 1979). The Calcein stained image series shows the cell survival timeline observed in both the mono- and co-cultured SCLC organoid models. In the monoculture SCLC organoid (Figure 3B), the SCLC tumor-like cell clusters are seen just before chemotherapy (0 DPT) but by 10 DPT, there are almost no visible live cells until about 38 DPT when the SCLC cell clusters regrow. This is quantified by fluorescence intensity (Figure 3D). On the other hand, the co-culture SCLC organoid (Figure 3C) also showed a large reduction in visible live cells by 10 DPT but there was never a complete disappearance of live cells and large SCLC cell clusters were seen by day 31 post therapy. We further examined the mono- and co-culture SCLC organoid models for cell viability using the Cell titre glo assay (Figure 3E). This quantification showed the same cell survival behavior as the fluorescence quantification from the CalceinAM live cell imaging. We also observed that the tumors co-cultured with ALFs showed significantly (*p* < 0.0001) higher cell viability post chemotherapy, which could result from ALF resistance to chemotherapy and/or increased SCLC cell resistance in the presence of ALFs.

To further investigate this striking difference in cell survival post-chemotherapy between mono- and co-culture SCLC organoid models, we examined the acute inflammatory factors secreted into the media that are typically associated with lung cancer progression, metastasis and angiogenesis (Cheng et al., 2016; Zhaofeng Tan et al., 2021; Parvez Khan, 2022). Cell culture supernatants secreted by both types of SCLC organoid models were collected at 0 DPT and 31 DPT and analyzed for secreted cytokines (interleukin (IL), IL-6, IL-8), chemokines [growth regulated alpha protein (GRO-α), monocyte chemoattractant protein-1 (MCP-1), vascular endothelial growth factor (VEGF-A)], and matrix metalloproteinases (MMP1, MMP2).

For both the 0 DPT and 31 DPT timepoints, all the inflammatory secreted factors tested except VEGF-A were present in significantly higher amounts in the co-culture SCLC model than in the monoculture SCLC model (Figures 3F–L). But as the SCLC monoculture organoid models can produce all these secreted factors at low level, we next asked whether the direct interaction of the ALFs with the SCLC cells could induce the SCLC cells to produce more of these factors, which could promote SCLC progression in the SCLC co-culture organoid models. We, therefore, next investigated how the ALF’s promote SCLC cell survival after chemotherapy in the co-culture SCLC organoid models.

### 2.4 Secreted paracrine factors from lung fibroblasts drive the regrowth of SCLC cells in the co-cultured organoid model

**FIGURE 4:**
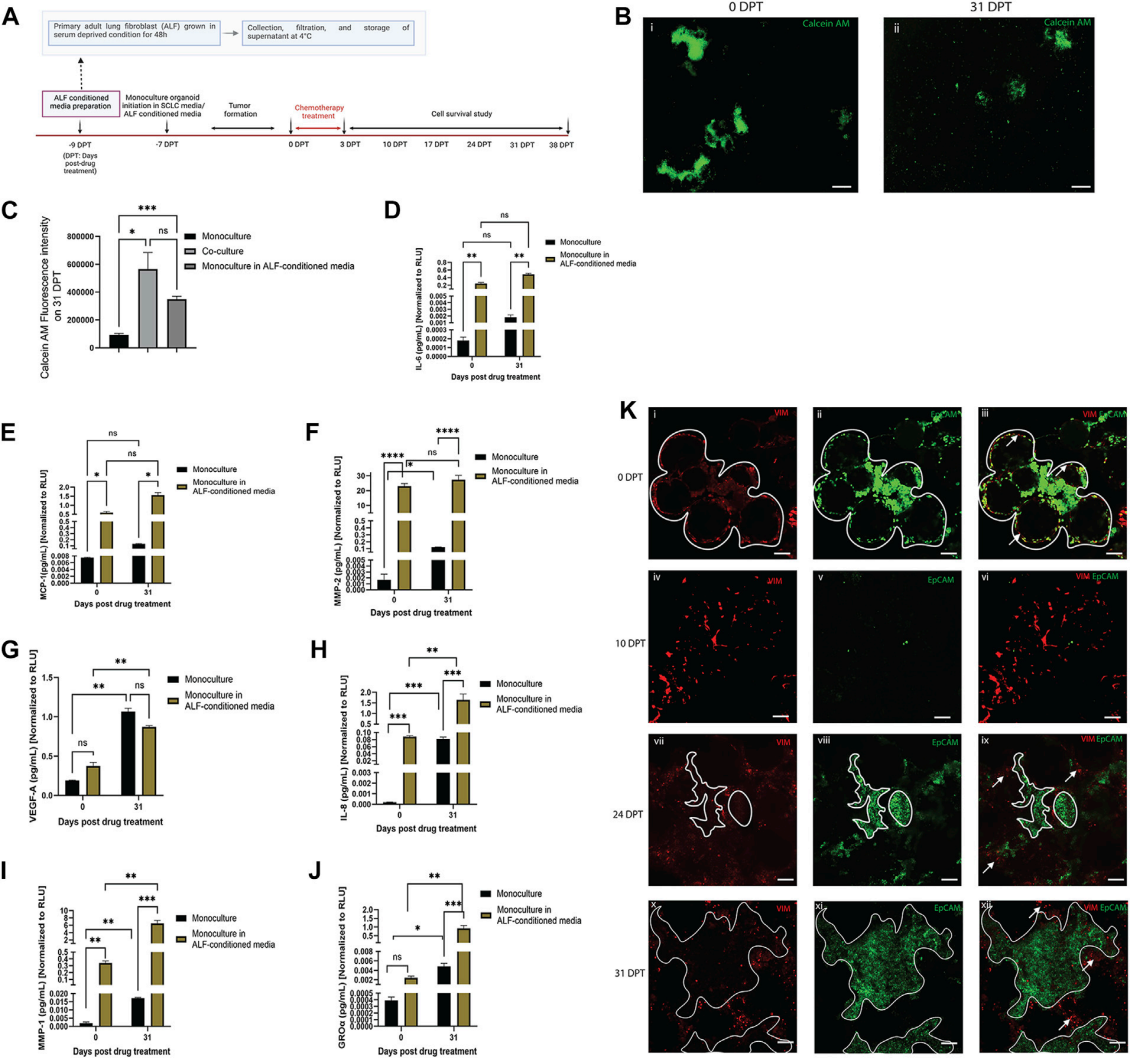
Effect of ALF-conditioned media on cell survival, cell regrowth and secreted factors in SCLC monoculture organoid model: **(A)** experimental timeline. **(B)** Fluorescent images of Calcein-AM stained live cells in monoculture organoid in ALF-conditioned media before drug treatment (0 DPT) and after cell regrowth (31 DPT) (scale bar: 500 µm). **(C)** Quantification of Calcein AM fluorescence intensity of monoculture, monoculture in ALF-conditioned media and co-culture organoid on 31 DPT; bar graph represents SEM; n = 3. *p* < 0.05, ∗∗∗*p* < 0.001 by one-way ANOVA. **(D–J)**: Quantification of acute inflammatory factors secreted by the monoculture organoid before drug treatment (0DPT) and after cell regrowth (31 DPT). Secreted factor values were normalized against RLU values (0 and 31 DPT) of monoculture organoid and monoculture organoid in ALF-conditioned media before plotting. Bar graph represents SEM; n = 3. ∗*p* < 0.05, ∗∗*p* < 0.01, ∗∗∗*p* < 0.001 by two-way ANOVA multiple comparison test. **(K)** Location of ALFs and SCLC cells before drug treatment (0DPT) and during cell regrowth after chemotherapy (10–31 DPT) (scale bar: 200 µm). White lined area shows tumor boundary and white arrows show regions enriched in ALF. At 0 DPT, both cell populations were closely interacting **(Ki–iii)** but post-chemotherapy, during 24–31 DPT, with rapid tumor growth, the ALFs are mostly pushed to the tumor boundary and not integrated in the tumors **(Kvii–xii)**.

In order to better understand whether regrowth of SCLC cells after chemotherapy may be driven by paracrine factors from healthy adjacent fibroblasts, we used monoculture organoid models (SCLC cells only) cultured in either ALF-conditioned media (derived from ALFs grown in 2D cultures in serum deprived media) or in monoculture media, and treated with Cisplatin and Etoposide, as described before (Figure 3A). In both cases, the monoculture organoid models were cultured for 31 days after chemotherapy and the inflammatory factors secreted in both conditions were examined from time points before treatment (0 DPT) and at 31 days after treatment (31 DPT). The experimental details and timelines are shown in Figure 4A. We used Calcein AM cell-permeant dye imaging to demonstrate the phenotype of the SCLC monoculture organoid models in either ALF-conditioned media or monoculture media before drug treatment (0 DPT) and post recurrence (31 DPT) (Figure 4B). Comparing the live cell intensity (using Calcein AM) profile of monoculture (in monoculture media), monoculture in ALF-conditioned media, and ALF-SCLC co-culture organoid models (Figure 4C) on 31 DPT, we found that monoculture organoids in ALF-conditioned media showed a significantly higher live cell intensity than monoculture organoids in monoculture media and this level of intensity was similar to that of the co-cultured organoids. This suggests that in the presence of ALF-conditioned media, the monoculture organoid models show higher cell regrowth post-chemotherapy than in monoculture media.

To validate this phenotypic observation, next we compared the secreted inflammatory factors of the two test conditions and, as elucidated in Figures 4D–J, SCLC monoculture organoids in ALF-conditioned media produced higher levels of the secreted inflammatory factors than those in monoculture media except VEGF-A. Because the same batch of ALF-conditioned media was used throughout the whole experiment, we next compared levels of each inflammatory factor secreted by the monoculture organoid models in ALF-conditioned media at 0 and 31 DPT. Comparison of each factor before and after SCLC cell regrowth revealed that GRO-α, IL-8 and MMP-1 had significantly higher expression in the media with SCLC cell recurrence than in the original tumor before chemotherapy (Figures 4H–J). This suggests that the ALF-conditioned media induced the SCLC cells to secrete more GRO-α, IL-8, and MMP-1 and these factors all play key roles in angiogenesis and tumor metastasis (Zhu et al., 2004; Morishita et al., 2018; Shengnan Yu et al., 2020). Interestingly, the SCLC monoculture organoids cultured in monoculture media showed a trend towards secreting more GRO-α, IL-8, MMP-1 and MMP-2 at DPT 31 than at DPT 0, but to a much smaller extent than the co-cultures, showing that SCLC cells secrete these factors when they survive after chemotherapy, but more so in the presence of ALFs.

We further examined the location of the ALF and SCLC cells in our co-culture model during chemotherapy and regrowth (0–31 DPT) to examine the proximity of these cells for cell-cell interactions (Figure 4K). Vimentin-expressing ALFs (red) and EpCAM-expressing SCLC cells (green) were initially patterned in close opposition to each other (0 DPT) surrounding the bead scaffolds with SCLC cells being the dominant population (Figures 4Ki–iii). Post-chemotherapy (10 DPT), there is disruption of the SCLC organoid model structure with some visible ALFs and a few surviving SCLC cells (Figures 4Kiv–vi). With time in culture, (24–31 DPT), the SCLC population takes over the culture again with only a few remaining fibroblasts and these are found mostly on the periphery of the tumor regions (Figures 4Kvii–xii). Therefore, at least in the context of cell survival after chemotherapy, we surmise that the paracrine effects from ALFs contribute more to SCLC cell survival after chemotherapy than direct SCLC cell-ALF interactions.

## 3 Discussion

Here, we have developed a scaffold-based 3D co-culture SCLC organoid model using a top-down approach. The addition of SCLC cells to the ALFs in the lung organoid model microarchitecture reveals the phenotypic transformation and kinetics of *in vitro* tumor formation and also allows the study of cell-cell interactions and paracrine effects in the model. The immunofluorescent staining of the SCLC co-culture organoids reveals the expression of neuroendocrine biomarkers that are classic of the disease. The SCLC organoid co-culture model can also be used to assess microenvironmental parameters like mechanobiological changes over time in culture. Stiffening of tumors is reported to be a sign of tumor microenvironment remodeling with tumor cell growth, displacement of host tissue, and cancer cell invasion of surrounding tissues (Zaman et al., 2006; Gkretsi and Stylianopoulos, 2018; Maria and Stylianopoulos, 2018; Ramamoorthy et al., 2019). We observed increasing tumor stiffness and displacement of the scaffold with time in culture suggesting that this co-culture model could be useful for analyzing the mechanobiological changes that occur with cancer progression.

Relevant SCLC models that mimic the tumor relapse seen in patients are challenging to develop because the tumor microenvironment is key for developing this phenotype. We generated a scalable 3D SCLC co-culture model in the dish that can be used to study this biology and therefore has potential to be used for high throughput drug screening (HTS). We used the SCLC organoid model to study the role of ALFs in SCLC co-culture organoids and the model lends itself to the addition of other cell types to further understand the role of the microenvironment in disease progression and relapse. The model allows the integration of different SCLC cell lines as well as inclusion of different types of fibroblasts, such as ALFs from different donors, or cancer associated fibroblasts. Our model was also able to distinguish between the effects of direct cell-cell interactions and paracrine signaling between ALFs and SCLC cells on tumor cell survival and this is beneficial for studying SCLC biology and has implications for therapeutic development.

Taken together, our study shows that we have built a powerful new tool for studying SCLC, that is, modular and allows the incorporation of multiple cell types and microenvironmental factors that influence tumor behavior. Currently, there is no available *in vitro* model of human SCLC that phenocopies the tumor microenvironment and demonstrates its effects on chemoresistance. Our SCLC organoid model can be used to answer in-depth biological questions on SCLC tumor development, progression, and relapse. The model’s phenotypic similarity with alveolar microarchitecture, scalability and its amenability to automated image analysis make it promising for a faster, more relevant, image-based phenotypic tool for HTS for resistant disease.

## 4 Materials and methods

### 4.1 Organoid generation

The organoid generation involved multiple steps that are explained in Section 4.1.1, Section 4.1.2, and Section 4.1.3.

#### 4.1.1 Scaffold generation and functionalization

The detailed method for alginate bead generation and functionalization is described in (Wilkinson et al., 2017). Briefly, the alginate beads (average diameter 100 µm) were generated using a custom-made electrostatic droplet generator operated at 9000 V. We used 3% alginate solution (Sigma-Aldrich A1112) as the biopolymer base running over a bath of 100 mM BaCl2 (Sigma-Aldrich 342920) as the crosslinking agent. Once the beads were made, we functionalized them in a two-step process. We soaked the beads with high-concentration rat tail collagen I solution (Corning 354249) for 6 days at 4°C followed by removal of excess collagen and coating with dopamine hydrochloride for 1 h at room temperature. Functionalized beads were then rinsed and soaked in experimentally relevant media at 4°C until further use.

#### 4.1.2 SCLC and ALF cell culture and media preparation

Human SCLC cell lines NCI-H526 (CRL-5811), NCI-H1963 (CRL-5982), and NCI-H82 (CRL-5811) were purchased from ATCC. After receiving the cells, they were cultured in standard tissue culture flasks (Genesee scientific) in the SCLC media [RPMI 1640 (Gibco), 10% fetal bovine serum (ATCC), 0.2% primocin] and cell passages <10 were used for this study.

Human primary adult lung fibroblasts (ALF) were isolated from distal lung tissue from a de-identified healthy donor (65-year-old, male, Caucasian, non-smoker, non-alcoholic) procured from the International Institute for the Advancement of Medicine (IIAM). Human lung tissue was procured under the UCLA approved IRB protocol #16–000742. The distal tissue was cut into 1 cm × 1 cm pieces and kept in 6-well plates, submerged in ALF media (DMEM + F12, 10% FBS, 1% non-essential amino acids, 1% glutamax) for 3–4 weeks to allow the fibroblasts to “crawl out” of the tissue and form an adherent mono layer in the well. The “crawled-out” populations were dissociated with TrypLE (Thermofisher 12605036), cultured in cell culture flasks and passages <5 were used for this study.

SCLC media is referred to as monoculture media and the combination of ALF and SCLC media in the same ratio as the cells (4:1) is referred to as co-culture media throughout this manuscript.

For the ALF-conditioned media preparation, a subculture of ALFs was cultured in ALF media without serum for ∼48 h in a separate flask until ∼80% confluency was achieved. The culture supernatant was then collected, filtered and used for the entirety of the experiment.

#### 4.1.3 Bioreactor setting, 3D model formation and loading into 96-well plate

To develop the 3D model, we used a high aspect ratio vessel (HARV) bioreactor vessel (model: RCCS-4H; Synthecon, Houston, Texas) of 2 ml volume and added 0.5 ml of functionalized microbeads and 1.5 ml of media containing a total of 1 million cells. The vessel was screwed into the bioreactor base and the beads and the cells allowed to settle. After sedimentation, the bioreactor was powered on to 4 rpm.

For the co-culture model, the optimized initial population ratio of ALFs and SCLC cells was 4:1, and the co-culture media was used. To mimic the initiation of SCLC disease, the ALFs and beads were added first in the bioreactor. Once the beads were coated with ALFs (∼6 h), representing a healthy normal lung, the SCLC cells were then introduced in the bioreactor and rotated until a uniform co-culture layering of cells was formed (∼48 h) that represented cancerous lung. For the monoculture model (SCLC cells only), 1 million SCLC cells with 0.5 ml beads and 1.5 ml monoculture media were rotated for 48 h. For the ALF-only model (Figure 1A), 1 million ALFs with 0.5 ml beads and 1.5 ml ALF media were rotated for 48 h. For all cellular combinations, after 48 h, the cell-coated bead solution was aliquoted 100 µl per well in a glass-bottom 96-well plate (Cellvis P96-1.5H-N) with the help of a multichannel pipettor. The 96-well plate was then briefly centrifuged (1000g, 2 min) to settle the cells/beads at the bottom of the plate and an additional 150 µl media was added to each well. The plate was then kept inside an incubator (37°C, 5% CO_2_, 95%RH) and monitored for the formation of self-organized 3D structures. Within the next 72 h, the fully-formed 3D models with micro-alveolar structures were observed in each well. From one bioreactor of 2 ml capacity, 20 organoids were formed. The number and capacity of the bioreactors were varied as required. For a full 96-well plate, 5 ml × 2 ml bioreactors or 1 ml × 10 ml bioreactor were used.

### 4.2 Atomic force microscopic analysis

For tumor stiffness measurements, 7-, 14- and 21-day old live 3D co-culture models were transferred to 35 mm fluorodishes (WPI, FD35-100) containing phosphate-buffered saline (PBS) buffer and atomic force microscopy was performed at 37°C in PeakForce Tapping mode using JPK Nanowizerd 4A (Bruker Nano Surface, CA, United States). We used a PeakForce Quantitative Nanomechanics- Live Cell (PFQNM-LC) probe (Bruker AFM probes, CA, United States) with a silicon tip [length = 54 µm; radius = 4.5 µm; frequency = 45 kHz; Spring constant = 0.1 N/m], specially optimized for soft biological samples. During measurements, multiple (n ≥ 3) tumor locations were selected and an area of 100 µm × 100 µm was scanned in each location. Force-distance curves were recorded to obtain tumor stiffness. Data analysis was done in JPKSPM Data processing software (version 6) (Bruker, United States). For calculating Young’s modulus, force-distance curves were converted to force-separation curves and Hertz-Sneddon model was chosen during model fitting.

### 4.3 Chemotherapy treatment and relapse study

For the chemotherapy treatment, Cisplatin (Tocris, 5B/266434) and Etoposide (Sigma, 099M4892V) were used. The drugs were added to the wells containing 7-day old SCLC tumors at a concentration equal to their respective half-maximal inhibitory concentration (IC50). To calculate the IC50, the tumors were treated with cisplatin and etoposide, singly and in combination, in a range of 0–100 µM, for 72 h. Upon reaching the end point, cell viability was determined via Cell-Titre-Glo assay (Promega) according to manufacturer’s protocol for viability determination. From the concentration and corresponding viability values, IC50 was calculated using Graphpad Prism (version 9) software. All treatments were done in triplicate.

To study cancer relapse after chemotherapy treatment, we measured cell viability by image analysis and using Cell-Titre-Glo kit (Promega) to detect metabolically active cells. All readings were taken in triplicate at 7-day intervals for a duration of 0–38 post-drug treatment days.

### 4.4 Imaging of live organoids and image analysis

For Supplementary Video S1, the ALFs were tagged with a vimentin promoter RFP reporter and SCLCs were tagged with an EpCAM promoter GFP reporter and the 3D rendering video was captured with a Leica Thunder confocal microscope.

For other live cell imaging, the live organoids were stained with Calcein-AM (Thermofisher, C3099) a GFP fluorescent live cell labeling dye at 1:1000 dilution, and imaged in a Zeiss Inverted Phase Contrast Fluorescence Microscope (Axiovert 40 CFL) at regular intervals (as per experimental design). For each time point and each treatment, experiments were done in triplicate. For image analysis, images of the same set of wells were captured weekly to monitor for change over time. Every time, fresh Calcein AM dye was added and any residual dye was washed off with media after the imaging was completed.

We used ImageJ (National Institute of Health, United States) software to analyze tumor area. For a particular image, the GFP zones were selected and the inbuild area measurement tool of the software was used to calculate area (J, 2007). The scale was converted from pixel to equivalent micron using the actual scale of the image.

For the intensity analysis of an image, we first computed a threshold to separate out the background from the foreground component of the image. In particular, we used the popular Otsu’s method (Otsu, 1979) to compute the intensity threshold *I*th. Otsu’s method instinctively performs clustering-based thresholding that assumes two classes of pixels backing bi-modal histogram (foreground and background pixels). For each image, we then compute the binary mask of dimension the same as that of the image, with logical ones corresponding to the pixel locations having value > *I*th. To compute the positive intensity, we then evaluate the mean of the masked image. Similarly, for negative intensity evaluation we compute the intensity of the complementary masked image. To expedite the intensity computation and allow automated intensity evaluation for an entire image batch, we developed a script in Python language. Additionally, the presented automated intensity analysis process may significantly reduce human error associated with image-wise manual intensity evaluation. We used Python language (version 3.7) to evaluate the intensity for the collected image set. The images were saved in grey scale. tif format with a resolution of 1269 × 972.

The example of intensity analysis is shown in Supplementary Figure S2.

### 4.5 Immunofluorescence staining and imaging of fixed organoid

For whole-mount staining, the organoids were first fixed using 4% paraformaldehyde (Thermo Fisher) for 30 min at room temperature and then permeabilized using 0.1% TritonX-100 (Sigma-Aldrich) in Tris-buffered saline (TBS) for 15 min. After blocking in DAKO (Agilent) for 1 h, organoids were incubated with primary antibodies overnight at 4°C. Next day, after triple washing, organoids were incubated in secondary antibodies (ThermoFisher) along with 4’, 6-diamidino-2-phenylindole (DAPI) for 1 h at room temp. The following primary antibodies were used: mouse anti-vimentin (Abcam), rabbit anti-calcitonin gene related peptide (CGRP) (Millipore Sigma), rabbit anti-EpCAM (Abcam), rabbit anti-synaptophysin (Abcam), mouse anti-chromogranin A (Proteintech), NCAM1 (Cell Signaling Technology). Confocal imaging was performed using Zeiss LSM 880.

### 4.6 Cell-culture derived xenograft (CDX)

Detailed protocol of CDX development is given in Shia et al., 2022. Briefly, 6–8 weeks old female NOD SCID gamma (NSG) mice (The Jackson Laboratory, 005557) were used for this study. H526 Cells from standard 2D culture in tissue culture flask were used as SCLC tumor source. Cells were dissociated and resuspended in a mixture of standard RPMI and Matrigel (Corning, 354234) prepared at a 1:1 ratio at a density of 1 × 107 cells/ml. 100 μl of cell suspension was injected subcutaneously into the right flanks of each mouse under isoflurane (Henry Schein, G125F19A). Mice were monitored daily for tumor growth. For this study, CDX tumor samples with no drug treated were used. Upon reaching endpoints, xenograft tumor samples were dissected and fixed in 4% formaldehyde overnight at 23°C. Samples were further incubated in a solution of 25% w/v sucrose dissolved in distilled H2O overnight at 4°C. Samples were paraffin embedded, sectioned at 4 μm thickness, and immune-stained. All animal studies were performed in compliance with ethical regulations and with approval from IUCAC.

### 4.7 Statistical analysis

All data were compiled from three or more independent replicates for each experimental condition. Data comparisons and statistical significance analysis were performed using one-way and two-way ANOVA using the GraphPad Prism software (version 9).

## Data Availability

The original contributions presented in the study are included in the article/Supplementary Material, further inquiries can be directed to the corresponding author.

## Supplementary material

SUPPLEMENTARY VIDEO S1 3D-rendering of a co-culture SCLC organoid model with small cell lung cancer (SCLC) cells (GFP tagged for EpCAM reporter) and adult lung fibroblast (RFP tagged for Vimentin reporter). For this video, the H526 cell line was used as the SCLC cell population in the model.mmc1.

SUPPLEMENTARY VIDEO S2 Movement of H526 cancer cells surrounding the microbeads in the SCLC co-culture organoid model.

SUPPLEMENTARY FIGURE S1 Effect of different seeding densities of ALF and SCLC on the model phenotype. ALF: SCLC 1:1 and 1:4 shows overpopulation of SCLCs. So, the selected seeding density for further experiments was ALF: SCLC- 4:1.

SUPPLEMENTARY FIGURE S2 Phenotypic representation of tumor growth in the SCLC co-cultured model using different SCLC cell lines: **(A)** H1963 and **(B)** H82; (scale bar: 500 µm).

SUPPLEMENTARY FIGURE S3 IC50 curve of different SCLC cell lines in the model.

SUPPLEMENTARY FIGURE S4 Automated image intensity analysis in the model.
