# Supplementary material for: Development of a small cell lung cancer organoid model to study cellular interactions and survival after chemotherapy
Source: Front Pharmacol. 2023 Aug 7;14:1211026. doi: 10.3389/fphar.2023.1211026 (PMC10441219; doi:10.3389/fphar.2023.1211026)
Supplement: Supplementary file 1 [file Presentation1.PPTX]

## Slide 1
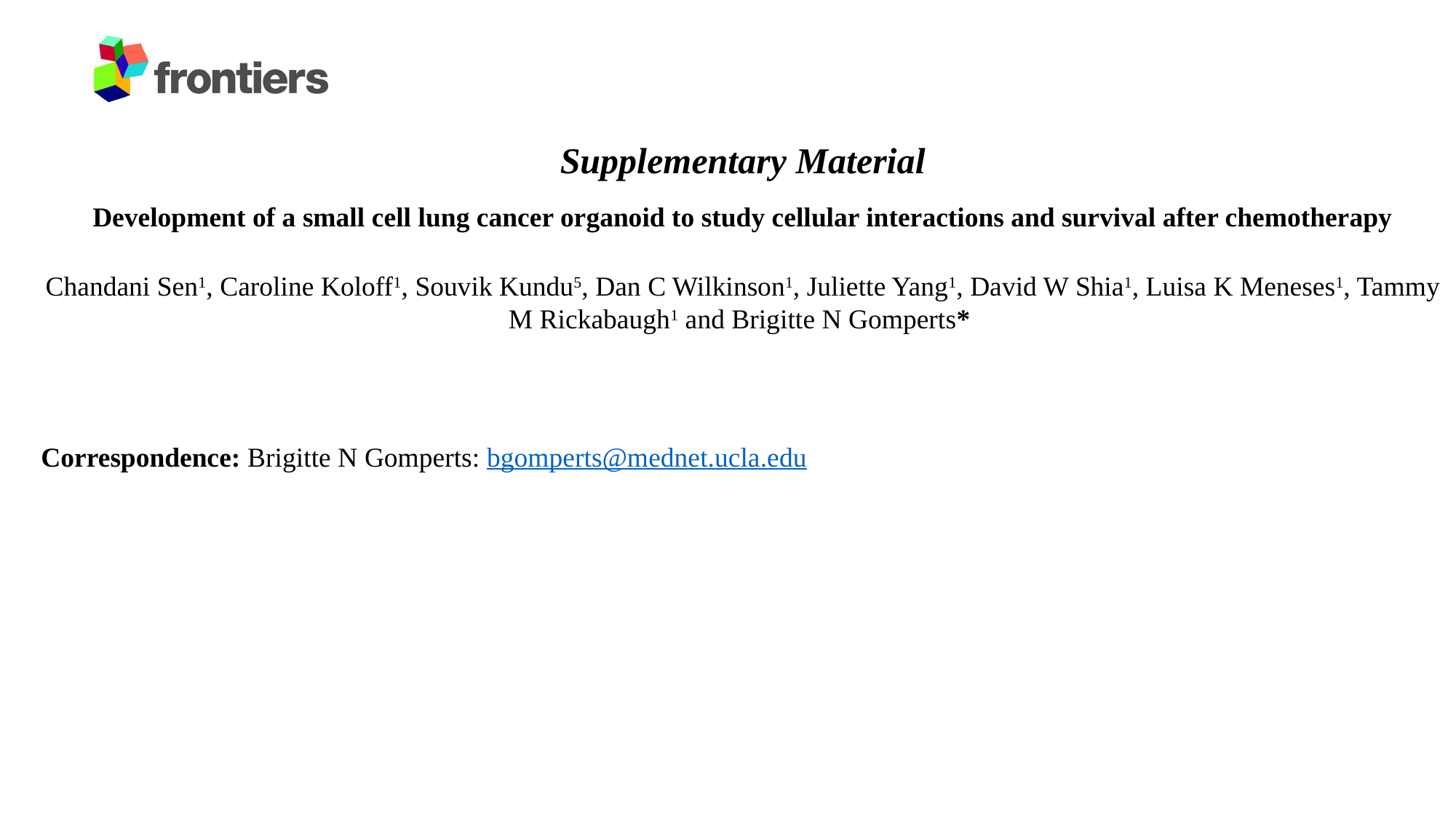

Supplementary Material
Development of a small cell lung cancer organoid to study cellular interactions and survival after chemotherapy
Chandani Sen1, Caroline Koloff1, Souvik Kundu5, Dan C Wilkinson1, Juliette Yang1, David W Shia1, Luisa K Meneses1, Tammy M Rickabaugh1 and Brigitte N Gomperts*
Correspondence: Brigitte N Gomperts: bgomperts@mednet.ucla.edu

## Slide 2
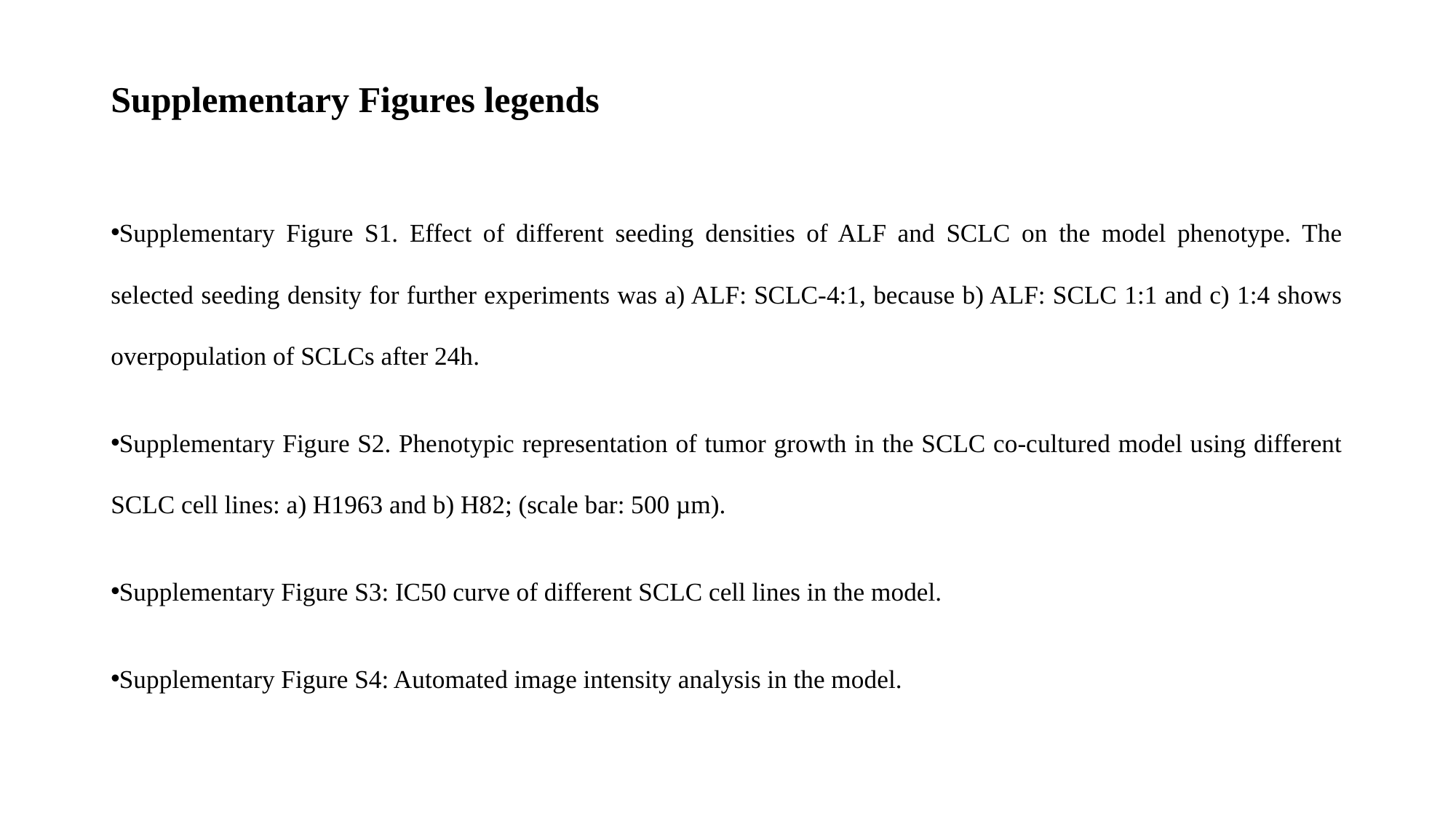

# Supplementary Figures legends
Supplementary Figure S1. Effect of different seeding densities of ALF and SCLC on the model phenotype. The selected seeding density for further experiments was a) ALF: SCLC-4:1, because b) ALF: SCLC 1:1 and c) 1:4 shows overpopulation of SCLCs after 24h.
Supplementary Figure S2. Phenotypic representation of tumor growth in the SCLC co-cultured model using different SCLC cell lines: a) H1963 and b) H82; (scale bar: 500 µm).
Supplementary Figure S3: IC50 curve of different SCLC cell lines in the model.
Supplementary Figure S4: Automated image intensity analysis in the model.

## Slide 3
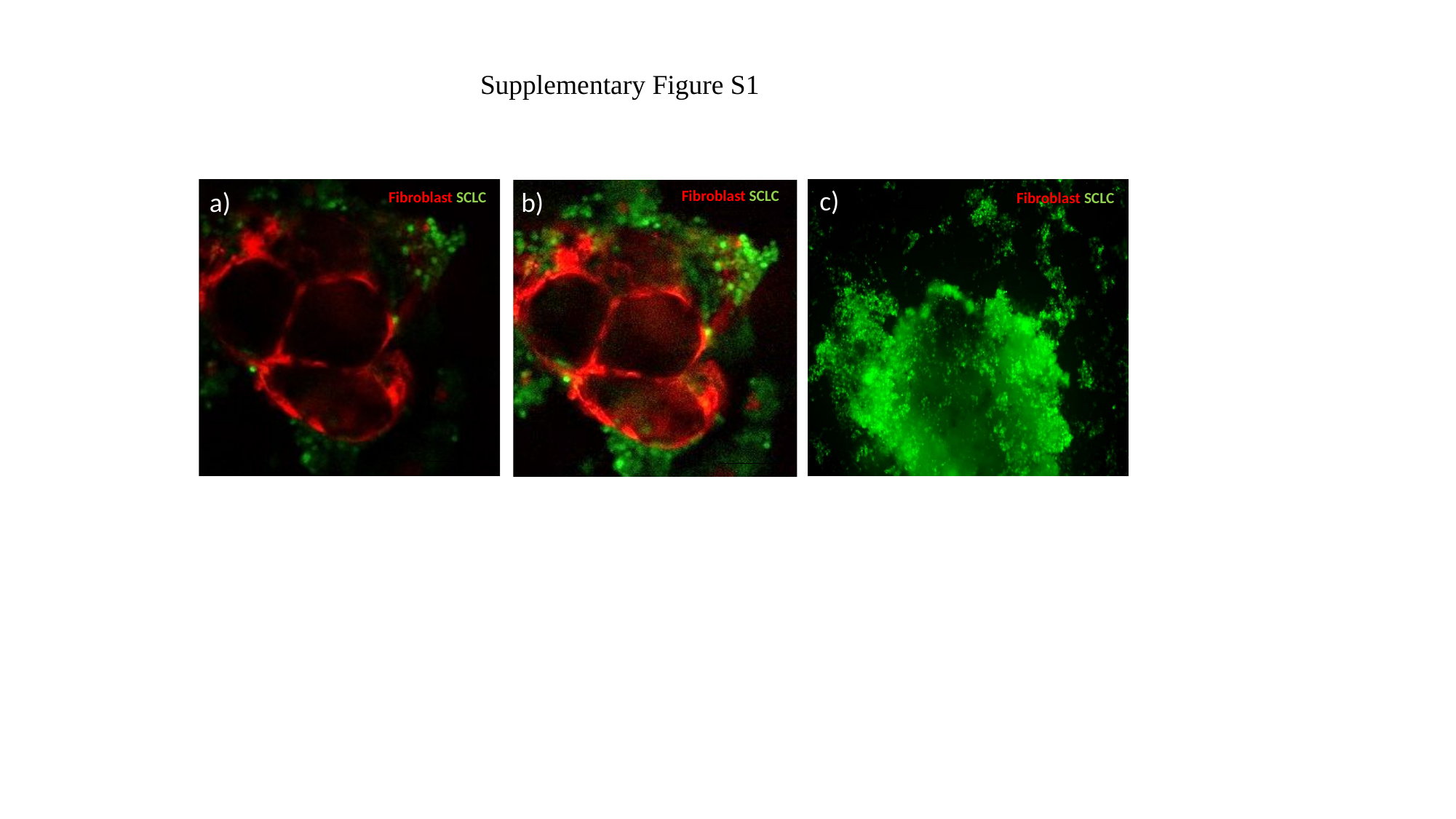

Supplementary Figure S1
c)
Fibroblast SCLC
SCLC tumor phenotype after 7days
Fibroblast SCLC
Fibroblast SCLC
a)
b)

## Slide 4
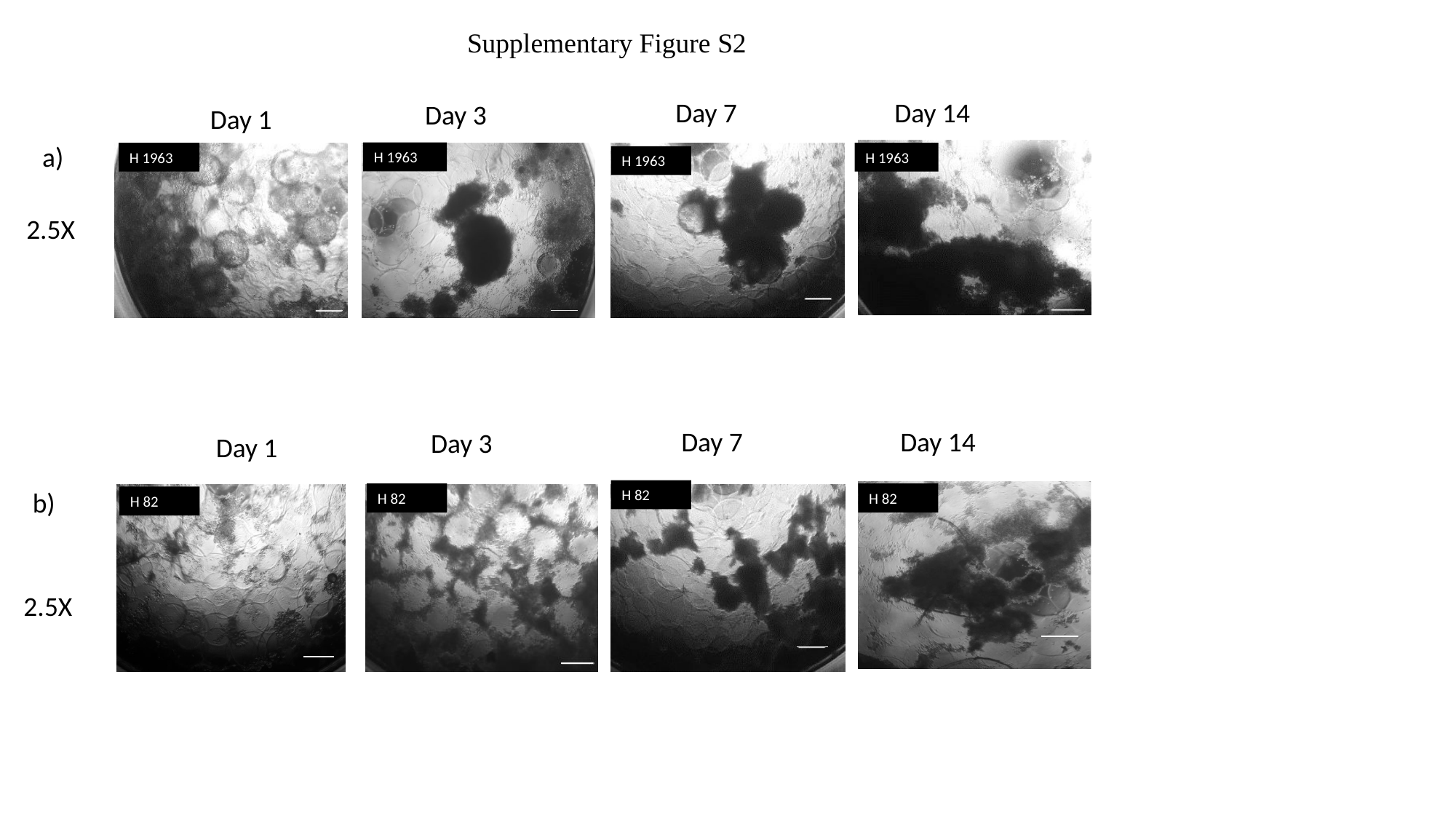

Supplementary Figure S2
Day 7
Day 14
Day 3
Day 1
a)
H 1963
H 1963
H 1963
H 1963
2.5X
Day 7
Day 14
Day 3
Day 1
b)
H 82
H 82
H 82
H 82
2.5X

## Slide 5
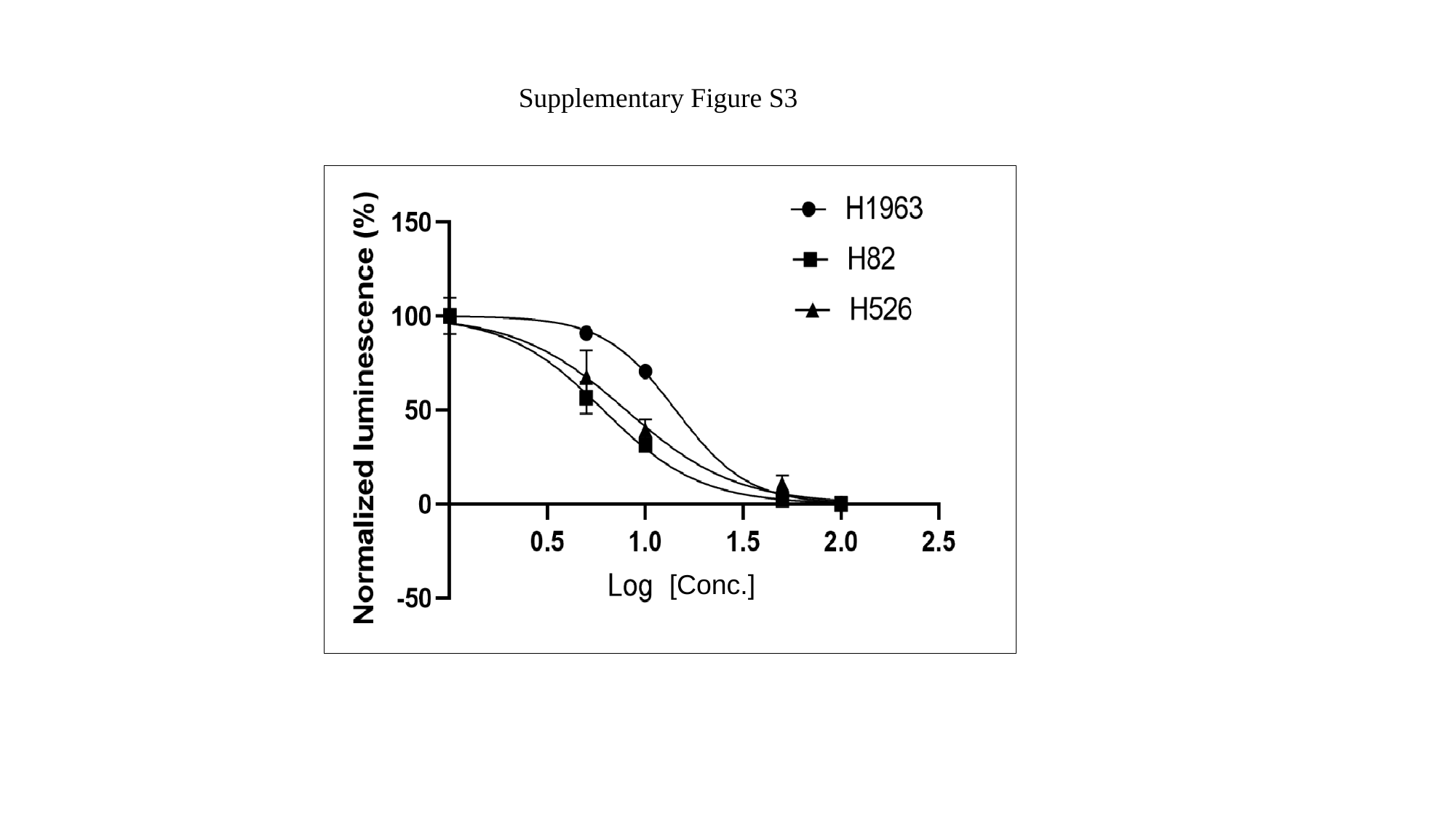

Supplementary Figure S3
[Conc.]

## Slide 6
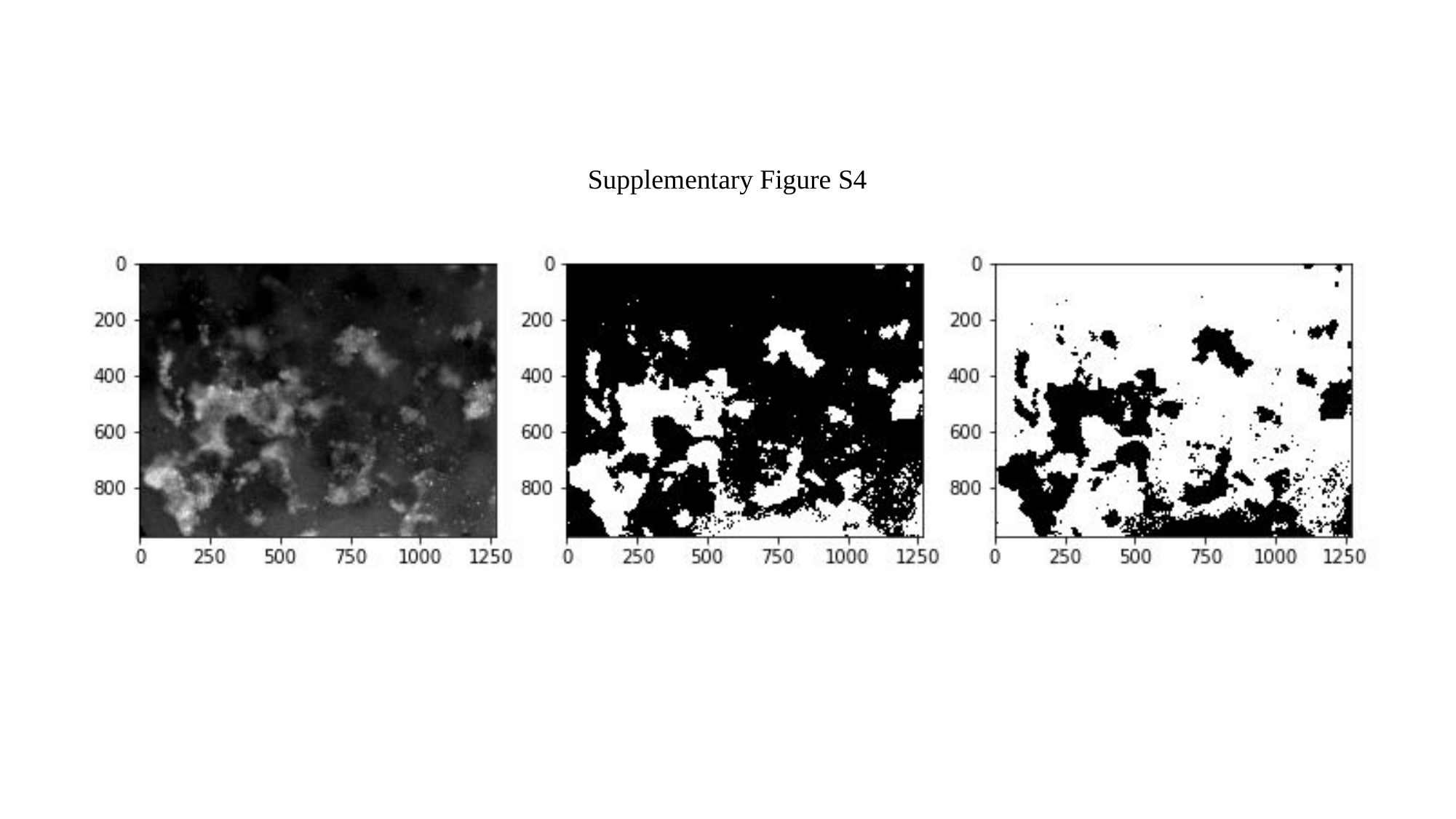

Supplementary Figure S4

## Slide 7
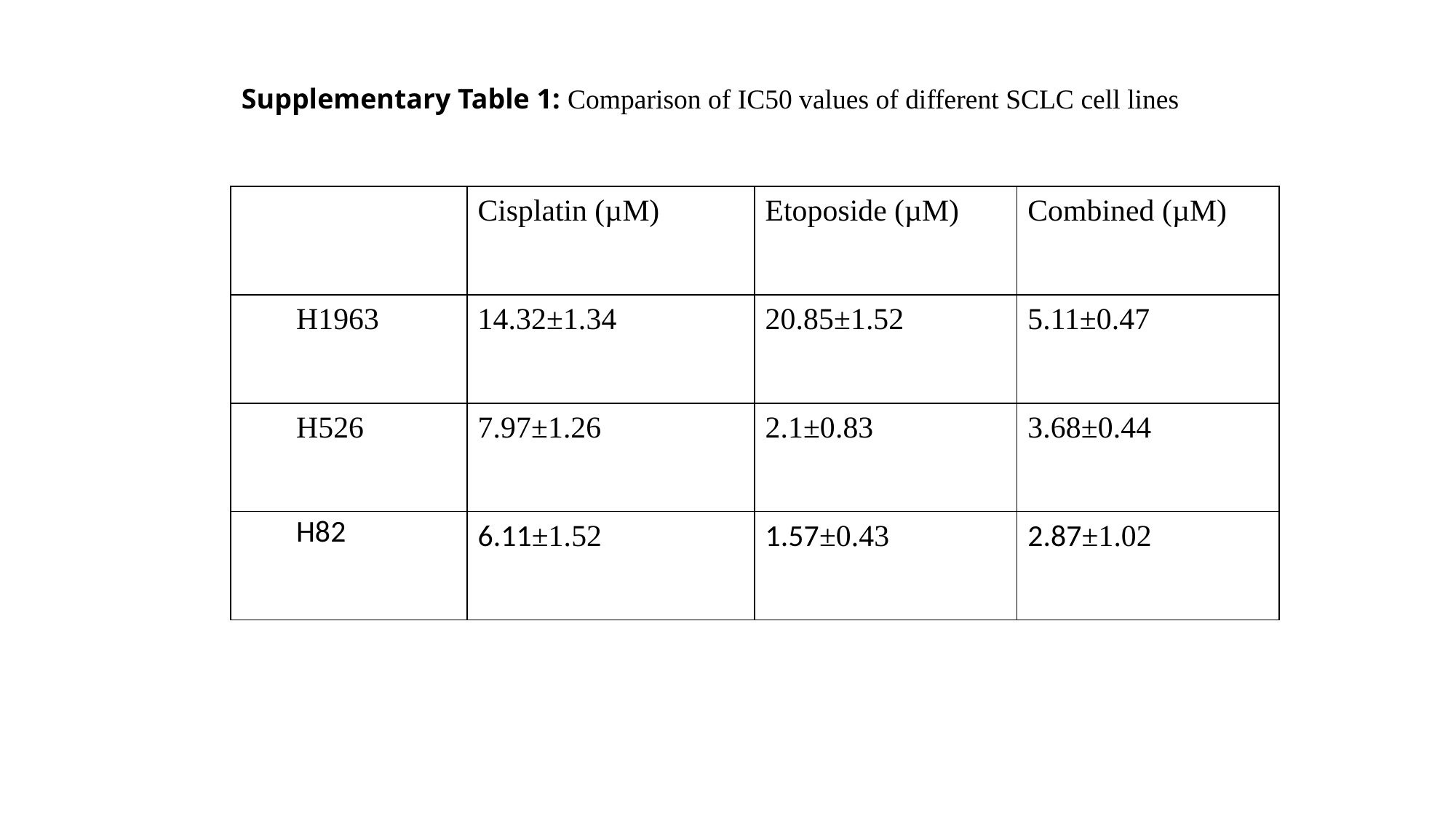

Supplementary Table 1: Comparison of IC50 values of different SCLC cell lines
| | Cisplatin (µM) | Etoposide (µM) | Combined (µM) |
| --- | --- | --- | --- |
| H1963 | 14.32±1.34 | 20.85±1.52 | 5.11±0.47 |
| H526 | 7.97±1.26 | 2.1±0.83 | 3.68±0.44 |
| H82 | 6.11±1.52 | 1.57±0.43 | 2.87±1.02 |
